# Protective Effects of *Lindera coreana* on UVB-induced Oxidative Stress in Human HaCaT Keratinocytes

**Published:** 2014

**Authors:** Jia-Le Song, Yang Gao

**Affiliations:** a Department of Nutrition and Food Hygiene, School of Public Health, Guilin Medical University, People’s Republic of China.; b Department of Food Science and Nutrition, Pusan National University, Busan 609-735, South Korea.; c Department of Pharmacy, Northern Jiangsu People’s Hospital Affiliated to Yangzhou University (Clinical Medical College of Yangzhou University), Yangzhou, Jiangsu 225001,People’s Republic of China.

**Keywords:** *Lindera coreana*, HaCaT keratinocytes, Ultraviolet (UV), Reactive oxygen species (ROS), Antioxidant enzymes

## Abstract

The present study was designed to investigate the protective effect of ethanol extracts from *Lindera coreana* leaves (LCE) on UVB-induced oxidative stress in HaCaT keratinocytes. The HaCaT cells were pretreated with LCE for 24 h and then exposed to UVB (20 mJ/cm^2^) for 2 h. UVB significantly decreased the cell viability (p<0.05). LCE did not exhibit significantly cytotoxic effects and increased the viability of the HaCaT cells in a concentration-dependent manner. To further investigate the protective effects of LCE on UVB-induced oxidative stress in HaCaT cells, the cellular levels of reactive oxygen species (ROS), lipid peroxidation and endogenous antioxidant enzymes, including catalase (CAT), superoxide dismutase (SOD) and glutathione peroxidase (GSH-px), were analyzed. LCE decreased the intracellular levels of ROS and lipid peroxidation and increased the activity of antioxidant enzymes. These results suggest that LCE exerted cytoprotective activity against UVB-induced oxidative stress in HaCaT cells through the inhibition of lipid peroxidation, reduction of ROS levels and stimulation of antioxidant enzymes activities. In addition, LCE also decreased the TNF-α, IL-1β and IL-6 levels in UVB-irradiated HaCaT cells.

## Introduction

Ultraviolet (UV) radiation from the sunlight has been deemed as a potent environmental risk factor in non-melanoma skin cancer (1). It was also associated with the pathogenesis of sunburn cells, photoaging and acute inflammation in skin tissue (2). The UV light is divided into three categories depending on wavelength, long wave UVA (315-400 nm), middle wave UVB (280-315 nm), and short wave UVC (100-280 nm). In particular, UVB can cause the direct DNA damage, and/or indirect damage via production of reactive oxygen species (ROS) (3). Overproduced ROS can break the cellular pro-oxidant/antioxidant balance and leading to oxidative stress in keratinocytes and skin fibroblasts (4, 5). Generally, the mammalian cells have different antioxidative compounds, which including low-molecular-mass antioxidants such as glutathione (GSH), uric acid, vitamin C, vitamin E and various of endogenous antioxidant enzymes to against ROS-induced oxidative stress. As widely accepted, superoxide dismutase (SOD), catalase (CAT) and glutathione peroxidase (GSH-px) were three important endogenous antioxidant enzymes to against ROS-induced oxidative stress in living organs. As an essential cell, keratinocytes play an important role in the inflammatory response to UV irradiation of human skin (6). Chronic UVB irradiation was increased the production of some pro-inflammatory cytokines, such as tumor necrosis factor-alpha (TNF-α), interleukin (IL)-1β, IL-6 and IL-8, leading to the cell death in HaCaT keratinocytes (7-9).

Dietary botanical antioxidants supplement have been deemed showed protective effects against UVB-induced skin injury, and also inhibited the UVB-induced carcinogensis (10, 11). Some medicinal plants extracts were also showed a UV-light protective activity *in-vitro* (12). *Listea coreana *(also called hawk tea) is a traditional Chinese herb and widely distributed in many countries, especially in the southern part of China. It has been used as a folk medicine to prevent and treatment of gastrosis, hepatitis and some inflammatory diseases (13). Currently, some studies have reported that *Listea coreana* showed antioxidant, anti-virus, antidiabetic, hepatoprotective, hypolipidaemic, anti-inflammatory and immunomodulatory activity (14-18). However, whether LCE could protect HaCaT keratinocytes from UVB-induced oxidative damage has not been investigated. This study was designed to investigate the potential cytoprotective effects of LCE on UVB induced oxidative stress and also elucidated the mechanisms underlying its protective effects in HaCaT cells.

## Experimental

*Chemical reagents*

Dulbecco's modified Eagle medium (DMEM), fetal bovine serum, penicillin-streptomycin, 3-(4, 5-dimethylthiazol-2-yl)-2, 5-diphenyl tetrazolium bromid (MTT), and dihydrodichlorofluorescein (H_2_DCF-DA) were obtained from Sigma-Aldrich Co. (St. Louis, MO, USA). Dimethyl sulfoxide (DMSO), thiobarbituric acid (TBA), trichloroacetic acid (TCA) and pyrogallol were obtained from Tokyo Chemical Industry Co., LTD (Tokyo, Japan). All other reagents were analytical grade.

*Plant extracts preparation*

Fresh *Lindera coreana *leaves were purchased from a local market in Yangzhou, China in December 2011. The fresh *Lindera coreana *leaves were shade dried initially, freeze dried and then ground to a fine powder. A twelve-fold volume of ethanol (70%, v/v) was added to the powdered samples and extracted third by stirring overnight. *Lindera coreana* leaf ethanol extracts (LCE) were concentrated by heat evaporation and freeze-drying. LCE was redissolved in dimethyl sulfoxide (DMSO) at a concentration of 50 mg/mL, and stored at 4 °C until further study.

*Cell culture and UVB irradiation*

Human immortalized keratinocyte HaCaT cells were obtained from the China Center for Type Culture Collection (CCTCC, Wuhan, China). The cells were maintained in DMEM medium supplemented with 10% (v/v) fetal bovine serum (FBS) and 1% penicillin-streptomycinin in a humidified CO_2_ incubator (Model 3154, Forma Scientific Inc, Marietta, OH, USA) with 5% CO_2_ at 37 °C. When the cells were reached 90% confluence, they were washed twice with phosphate buffered saline (PBS) and then exposed to UVB for 2 h (20 mJ/cm^2^) with a UVB lamp (HP-30LM; Atto Co., Japan). To prevent light absorption by DMEM medium, the DMEM medium was removed just prior to irradiation and replaced with a thin layer of PBS to cover the cells. After irradiation, cells were fed with a DMEM growth medium. 

*Cell viability assay*

Cell viability was assessed by 3-(4, 5-dimethylthiazol-2-yl)-2, 5-diphenyl tetrazolium bromid (MTT) assay. Cells were seeded on 96-well plates at a density of 5 × 10^3 ^cells/well. After a 24 h incubation, the cells were treated with LCE (10-200 ug/mL) for 24 h, and then exposed to UVB for 2 h. Following UVB irradiation, 100 μL MTT reagent (0.5 mg/mL) was added to each well and the cells were incubated in a humidified incubate at 37 °C to allow the MTT to be metabolized. After 4 h, the medium was removed and the cells were resuspended in formazan with 100 μL DMSO. The absorbance of the samples was measured at 540 nm by microplate reader (Bio-Rad, model 680, Hercules, CA, USA).

*Analysis* *of intracellular ROS*

Intracellular ROS levels were measured using the fluorescent probe dihydrodichlorofluorescein (H_2_DCF-DA). Following treatment, the HaCaT cells were washed with calcium- and magnesium-free PBS and incubated in H_2_DCF-DA (20 μM) containing serum- and phenol-red-free DMEM medium for 30 min. After incubation, the medium was removed and the cells were washed twice with PBS. Fluorescence was measured using a FLUOstar OPTIMA fluorescence plate reader (BMG Labtec, Ortenberg, Germany; excitation was read at 485 nm and emission at 535 nm). Relative ROS production (percentage of the control) was expressed as the ratio of fluorescence of the treated samples over the response in the appropriate controls: (fluorescence _treatment_/fluorescence _control_) × 100.

*Lipid peroxidation levels*

Lipid peroxidation was evaluated by thiobarbituric acid reactive substance (TBARS) assay (19). In brief, the treated cells were washed with cooled PBS, scarped into TCA (2.8%, w/v) and sonicated, total protein was determined by bicinchoninic acid (BCA) assay. The suspension was mixed with 1 mL TBA (0.67%, w/v) and 1 mL TCA (25%, w/v), heated (30 min, 95 °C) and centrifuged (1,500 rpm, 10 min, 4 °C). TBA reacts with the oxidative degradation products of lipids to yield red complexes that absorb at 535 nm. The amount of TBA reactive substance was determined using UV-2401PC spectrophotometer (Shimadzu, Kyoto, Japan).

*Antioxidant enzymes activities*

HaCaT cells grown in 10-cm cell culture dish were pretreated with LCE (10-200 ug/mL) for 24 h and then exposed to UVB for 2 h for further analysis. The cells were washed with PBS, detached by scraping and centrifuged, and the resulting cell pellet stored at -80 °C. Cell pelltes were thawed, resuspended in 300 μL cold lysis buffer (PBS, 1mM EDTA), homogenized and centrifuged (1,200 rpm, 10 min, 4 °C). The resulting supernatants were used for activity measurements. CAT activity (U/mg protein) was according to the method described by Nelson and Kiesow (20) in which the disappearance of the substrate H_2_O_2_ was measured spectophotometrically at 240 nm. SOD activity (U/mg protein) was assayed using a modified autoxidation of pyrogallol method (21). One unit of SOD activity was defined as the amount of enzyme that inhibited the rate of autoxidation rate of pyrogallol by 50%. GSH-px activity (U/mg protein) was assayed by according to the method of Hafemen *et al.* (22). Protein contents were determined by Bio-Rad protein assay kit according to the manufacturer’s instructions.

*Reverse transcription polymerase chain reaction (RT-PCR) assay*

Total RNA was isolated with Trizol reagent (Invitrogen, Carlsbad, CA, USA) and centrifuged at 12,000 rpm for 15 min at 25 °C following the addition of chloroform. Isopropanol was added to the supernatant at a 1: 1 ratio and the RNA was pelleted by centrifugation (12,000 rpm for 15 min). After washing with ethanol, the RNA was solubilized in diethyl pyrocarbonate-treated RNase-free water and quantified by measuring the absorbance at 260 nm using a UV-2401PC spectrophotometer (Shimadzu, Kyoto, Japan). The primers for SOD, CAT, GSH-px and β-actin were as follows: SOD sense, 5’- ATGGCGACGAAGGCCGTGTG-3’, and antisense, 5’-GACCACCAGTGTGCGGCCAA-3’; CAT sense, 5’-CCTTCGACCCAAGCAACATG-3’, and antisense, 5’-CGAGCACGGTAGGGACAGTTC-3’; GSH-px sense, 5’-CCTGTACCAGTCCAATACCATCCT-3’, and antisense, 5’-TCCTGCTGGTCCTTCCCATA-3’; β-actin sense, 5’-TCCTTCTGCATCCTGTCGGCA-3’, and antisense, 5’-CAAGAGATGGCCACGGCTGCT-3’. Equal amounts of RNA (1 µg) were reverse transcribed in a master mix containing 1 × reverse transcriptase buffer, 1 mM dNTPs, 500 ng of oligodT_18_ primers(Invitrogen), 140 U of MMLV reverse transcriptase(Invitrogen), and 40 U of RNase inhibitor for 45 min at 42 °C. PCR was then carried out in an automatic thermocycler (Bioneer, Daejeon, South Korea) for 25 cycles (94 °C for 30 s, 60 °C for 30 s, and 72 °C for 40 s) followed by an 8-min extension at 72 °C. The PCR products were separated in 2% agarose gels and visualized by EtBr staining. β-actin was used for normalization. 

*Measure of cytokines concentration*

Pro-inflammatory cytokines (TNF-α, IL-1β and IL-6) productions were measured with an ELISA assay. HaCaT cells grown in 6-cell culture plates were pretreated with LCE (10-200 ug/mL) for 24 h and then exposed to UVB for 2 h. After UVB irradiation, aliquots of samples (100 uL/well) were collected from the experimental medium, and the cytokines (TNF-α, IL-1β and IL-6) productions were measured using a commercially available ELISA kit (R&D Systems, Minneapolis, MN, USA) according to the manufacturer’s instructions. 

*Statistical analysis*

Data are presented as the mean ± SD. Differences between the mean values for individual groups were assessed by a one-way ANOVA with Duncan’s multiple range tests. P<0.05 was considered to indicate a statistically significant difference. The SAS v9.1 statistical software package (SAS Institute Inc., Cary, NC, USA) was used for the analysis. 

## Results and Discussion

*Effects of LCE on UVB-induced oxidative damage in HaCaT cells*

**Figure 1:**
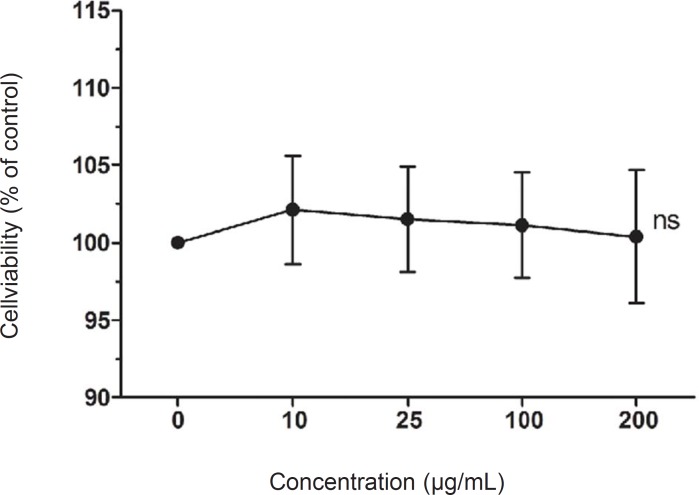
Effects of *Lindera coreana *leaf ethanol extracts (LCE) on cell viability in HaCaT keratinocytes. Data are representative of three independent experiments as mean ± SD. ^ns^ Means not significant

**Figure 2:**
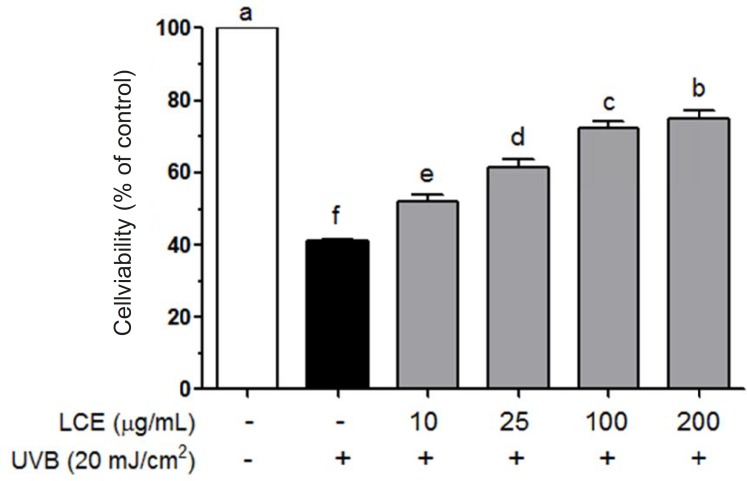
Effects of *Lindera coreana *leaf ethanol extracts (LCE) on cell viability in UVB (20 mJ/cm^2^) irradiated HaCaT keratinocytes. Data are representative of three independent experiments as mean ± SD. ^a~^^f^ Mean values with different letters on the bars are significantly different (p < 0.05) according to Duncan’s multiple range test

To investigate LCE-induced cytotoxicity, HaCaT cells were first treated with various concentrations of LCE (10-200 ug/mL) for 24 h and the cell viability was determined using MTT assay. LCE did not exhibited any significant cytotoxicity and the cell viability were both more than 90% (Figure 1). Based on these results, concentrations between 10 and 200 ug/mL were used to further studies. Human skin keratinocytes are essential cells in the skin and connective tissue, and are continuously exposed to UV irradiation (23). As shown in Figure 2, UVB (20 mJ/cm^2^) significantly induced the cell death in the HaCaT cells. However, following treatment with various concentrations of LCE, the cell viability was increased in a concentration-dependent manner. These results suggested that LCE exhibited a significantly protective effect which may be due to the free radical scavenging activity of LCE.

*Effects of LCE against UVB-induced intracellular ROS levels in HaCaT cells*

**Figure 3:**
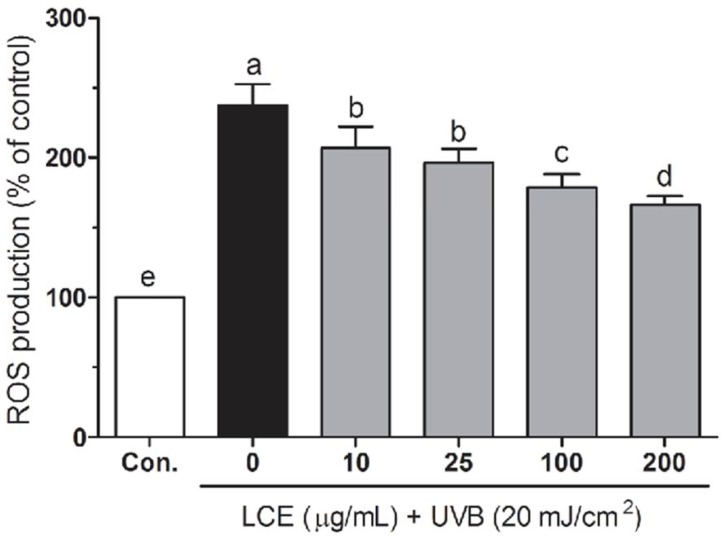
Effects of *Lindera coreana *leaf ethanol extracts (LCE) on intracellular reactive oxygen species (ROS) levels in UVB (20 mJ/cm^2^) irradiated HaCaT keratinocytes. Data are representative of three independent experiments as mean ± SD. ^a~^^e^ Mean values with different letters on the bars are significantly different (p < 0.05) according to Duncan’s multiple range test

UV-irradiation induced ROS generation plays a very important role in the skin aging, inflammation and photocarcinogengesis (24). Increased ROS can break the intracellular antioxidant defense system, resulting in some biomacromolecules (such as DNA, lipid and protein) damage and cause cell death (25). Botanical antioxidants have been reported that to attenuate ROS generation and reduce UV-induced skin photoaging and photocarcinogenesis (26, 27). To investigate the protective effects of LCE in UVB-irradiated HaCaT cells, the intracellular ROS levels were evaluated using a fluorescent probe H_2_DCF-DA. As shown in Figure 3, UVB significantly increased the ROS generation compared with those in the normal cells. In the presence of UVB, LCE significantly reduced the ROS generations in a concentration-dependant manner between 10 and 200 ug/mL. Treatment with same concentrations of LCE alone did not significantly increase the intracellular ROS levels (data not shown). These results suggested that LCE is a free radical scavenger. 

*Effects of LCE on lipid peroxidation in UVB-irradiated HaCaT cells*

**Figure 4:**
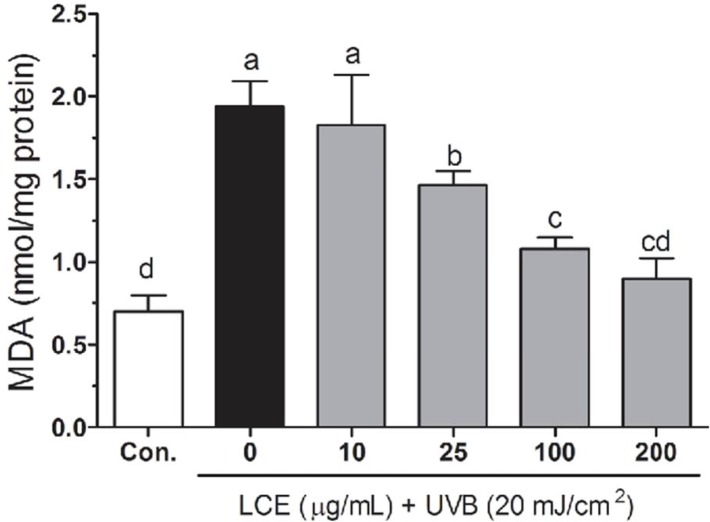
Effects of *Lindera coreana *leaf ethanol extracts (LCE) on intracellular malonaldehyde (MDA) levels in UVB (20 mJ/cm^2^) irradiated HaCaT keratinocytes. Data are representative of three independent experiments as mean ± SD. ^a~^^d^ Mean values with different letters on the bars are significantly different (p < 0.05) according to Duncan’s multiple range test.

Free radicals and ROS-induced oxidative stress were markedly associated with the lipid peroxidation of cell membranes and increased the levels of malondialdehyde (MDA), which is a biomarker of cell membrane lipid peroxidation. The increased MDA have been linked to damaging events such as loss of fluidity, inactivation of membrane enzymes, increased cell membrane permeability to ions, and finally rupture of cell membrane leading to release of cell organelles (28-30). As shown in Figure 4, increased lipid peroxidation levels were observed in UVB-irradiated HaCaT cells. However, treatment with the LCE resulted in a decrease in the lipid peroxidation generations, indicating that oxidative stress-related damage was lower in the LCE-treated HaCaT cells. The capacity of LCE to reduce lipid peroxidation may be due to its function as a preventive antioxidant to scavenge initiating radicals. 

*Effects of LCE on the activity of antioxidant enzymes in UVB-irradiated HaCaT cells*

**Figure 5:**
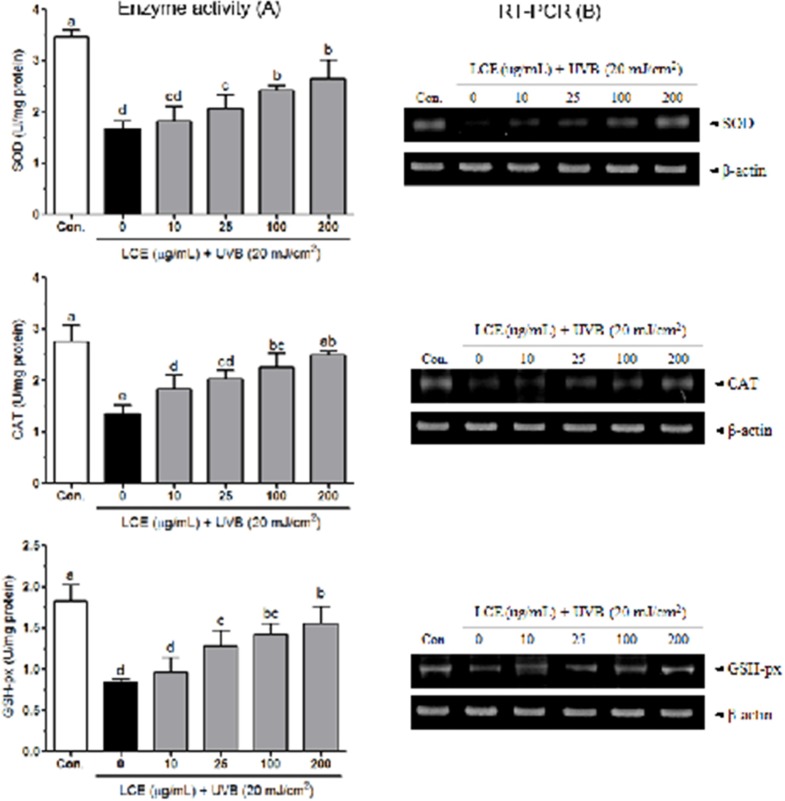
Effects of *Lindera coreana *leaf ethanol extracts (LCE) on the activity (A) and mRNA expression (B) of SOD, CAT and GSH-px in HaCaT cells exposed to UVB. Data are representative of three independent experiments as mean ± SD. ^a~^^e^ Mean values with different letters on the bars are significantly different (p< 0.05) according to Duncan’s multiple range test

Human keratinocytes contain high contents of antioxidants such as GSH and various endogenous enzymes involved in antioxidant defense, including SOD and catalase (31, 32). UVB significantly decreased the activities of SOD, catalase and GSH-px in HaCaT cells (33). In mammalian cells, overproduction of free radicals can be scavenged by endogenous antioxidant enzymes, including SOD, catalase and GSH-px. SOD catalyzes the conversion of superoxide (O_2_^-^) to hydrogen peroxide (H_2_O_2_) and H_2_O_2_ is further reduced H_2_O by the activity of catalase or GSH-px. SOD and catalase have been shown to protect human keratinocytes against UVB-induced cell damage (34-36). Figure 5A shows the intracellular antioxidant enzyme activities of LCE in UVB-irradiated HaCaT cells. The activity of SOD was reduced by UVB (to 1.68 ± 0.16 U/mg protein) compared with that in the normal cells (3.47 ± 0.13 U/mg protein), and this reduction was attenuated by various concentrations of LCE; the SOD activity was 1.82 ± 0.28, 2.07 ± 0.26, 2.43 ± 0.09 and 2.66 ± 0.35 U/mg protein at 10, 25, 100 and 200 μg/mL LCE, respectively. Following treatment with UVB, the cellular CAT activity was reduced (to 1.37 ± 0.17 U/mg protein) compared with that in the normal cells (2.75 ± 0.33 U/mg protein). However, the reduction in CAT activity was significantly attenuated (p<0.05) by treatment with LCE. The CAT activity was 1.84 ± 0.27, 2.03 ± 0.17, 2.26 ± 0.28 and 2.50 ± 0.08 U/mg protein at 10, 25, 100 and 200 μg/mL LCE, respectively. In addition, LCE also attenuated the UVB-induced reduction in GSH-px activity in the HaCaT cells. The GSH-px activity of the UVB-irradiated cells significantly increased following treatment with LCE; the increased levels ranged from 0.96 ± 0.18 to 1.56 ± 0.20 U/mg protein. In RT-PCR assay, UVB significantly reduced the mRNA expressions of SOD, CAT and GSH-px in HaCaT cells. Following treatment with various concentrations of LCE, the mRNA levels of those antioxidant enzymes were increased than that in the only UVB-irradiated HaCaT cells (Figure 5B). These results suggested that LCE treatment increased the activity of these antioxidant enzymes in the UVB-irradiated HaCaT cells, indicating that LCE was able to reduce UVB-induced oxidative stress.

*Effects of LCE on TNF-α, IL-1β and IL-6 levels in UVB-irradiated HaCaT cells*

TNF-α, IL-1 and IL-6 are produced and secreted by keratinocytes and play important roles in UVB irradiated photoaging, photoimmumology and apoptosis (37, 38). Some studies have suggested that UVB irradiation-induced the release of pro-inflammatory cytokines (TNF-α, IL-1β and IL-6) and led to the cell death in HaCaT keratinocytes (7, 9, 39). Dietary antioxidants can reduce the levels of TNF-α, IL-1β and IL-6 in UVB-irradiated mice (40, 41). As shown in Table 1, UVB significantly increased the levels of TNF-α, IL-1β and IL-6 compared with those in the normal cells. In the presence of UVB, LCE significantly reduces TNF-α, IL-1β and IL-6 levels in a concentration-dependent manner between 10 and 200 ug/mL. These results suggested that the protective effect of LCE may partly be associated with inhibiting the release of pro-inflammatory cytokines in UVB-irradiated HaCaT cells.

**Table 1:** Effect of LCE on the level of TNF-α, IL-1β and IL-6 in HaCaT cells exposed to UVB.

**Groups**	**Cytokine levels (pg/mL)**		
	**TNF-α**	**IL-1β**	**IL-6**
Normal	10.1 ± 1.5e1)	9.6 ± 1.4d	8.6 ± 1.3d
UVB	36.4 ± 4.8a	34.6 ± 4.5a	44.4 ± 5.8a
UVB + LCE (10 ug/mL)	34.6 ± 4.5a	32.9 ± 4.3a	42.2 ± 5.5ab
UVB + LCE (25 ug/mL)	30.9 ± 4.1bc	30.2 ± 3.2ab	37.8 ± 5.0b
UVB + LCE (100 ug/mL)	29.1 ± 3.8cd	26.2 ± 3.4b	36.6 ± 3.7b
UVB + LCE (200 ug/mL)	25.5 ± 3.3d	24.2 ± 3.2c	30.1 ± 4.8c

Data are representative of three independent experiments as mean ± SD.

a~e Mean values with different letters on the bars are significantly different (p<0.05) according to Duncan’s multiple range test. LCE: *Lindera coreana* leaf ethanol extracts; TNF-α: tumor  necrosis factor-alpha; IL-1β: interleukin-1β; IL-6: interleukin-6.

In conclusion, in the present study, LCE demonstrated the protective effect against UVB-induced cell death in human HaCaT keratinocytes. LCE was able to effectively scavenge UVB-induced ROS generations, decrease the lipid peroxidation of the cell membrane and prevent HaCaT cell death by increasing the activity of the intracellular antioxidant enzymes SOD, CAT and GSH-px. Furthermore, LCE also effectively reduced the UVB-irradiation induced pro-inflammatory cytokines (TNF-α, IL-1β and IL-6) increasing in HaCaT cells.
